# Sustainable Development for Mobile Health Apps Using the Human-Centered Design Process

**DOI:** 10.2196/45694

**Published:** 2023-08-25

**Authors:** Qingfan An, Marjorie M Kelley, Audra Hanners, Po-Yin Yen

**Affiliations:** 1 Department of Community Medicine and Rehabilitation Umeå University Umeå Sweden; 2 College of Nursing The Ohio State University Columbus, OH United States; 3 Institute for Informatics Washington University School of Medicine in St Louis St Louis, MO United States

**Keywords:** mHealth, mobile health, apps, human-centered design, sociotechnical, sustainability, mobile technology, speculative design, mobile phone

## Abstract

Well-documented scientific evidence indicates that mobile health (mHealth) apps can improve the quality of life, relieve symptoms, and restore health for patients. In addition to improving patients’ health outcomes, mHealth apps reduce health care use and the cost burdens associated with disease management. Currently, patients and health care providers have a wide variety of choices among commercially available mHealth apps. However, due to the high resource costs and low user adoption of mHealth apps, the cost-benefit relationship remains controversial. When compared to traditional expert-driven approaches, applying human-centered design (HCD) may result in more useable, acceptable, and effective mHealth apps. In this paper, we summarize current HCD practices in mHealth development studies and make recommendations to improve the sustainability of mHealth. These recommendations include consideration of factors regarding culture norms, iterative evaluations on HCD practice, use of novelty in mHealth app, and consideration of privacy and reliability across the entire HCD process. Additionally, we suggest a sociotechnical lens toward HCD practices to promote the sustainability of mHealth apps. Future research should consider standardizing the HCD practice to help mHealth researchers and developers avoid barriers associated with inadequate HCD practices.

## Introduction

Mobile health (mHealth) is defined by the World Health Organization [1] as the practice of medical and public health supported by mobile devices [2]. As smartphone use becomes ubiquitous, the increasing popularity of mHealth apps is not surprising. mHealth apps can address many limitations of traditional medicine. For example, mHealth apps can monitor real-time conditions, assess progress, improve communication, detect illness, help prevent disease exacerbations, manage and track chronic disease, and deliver therapeutic interventions at a convenient time to the patient [3-5]. Additionally, mHealth apps are increasingly used to reduce the burden of disease linked with poverty [6]. Most recently, the COVID-19 pandemic has intensified the focus of the development of mHealth apps [7] potentially due to quarantine policies and the large amount of health resources allocated to COVID-19 prevention and treatment [8]. Currently, patients and health care providers (HPs) have a wide variety of choices among commercially available mHealth apps. However, the cost-benefit appraisal of mHealth apps has sparked debate due to the high cost of resources and well-documented low acceptance by users [9]. Many mHealth apps are thought to lack a rigorous research process to back up their intervention efficacy, which may explain their low acceptance [10]. Furthermore, most research has focused on the technology rather than the service delivery and app content from the user’s perspective [11]. Most mHealth apps created from existing structures of health care systems or based on behavior change theories are assumed to be “one-size-fits-all” interventions [12]. Such apps often lack resultful end-user inputs, which may not be as effective as those that have users in mind [9,13-15]. Emerging research suggests that human-centered design (HCD) in mHealth can help address patient needs in the development process, which could improve user acceptance. mHealth is also expected to show effectiveness in improving patient outcomes if mHealth apps are sustained with long-term and regular use. The sustainability of mHealth use is a long-lasting issue. A need exists for guidance in the development of sustainable mHealth apps [16] as currently no standardized HCD guidance exists for mHealth app developers. mHealth studies provided limited or scattered reports on their HCD practices. There is still a lack of consensus regarding the HCD practice when developing mHealth apps [17]. A discussion on evidence-informed or evidence-based HCD practice could aid in the sustainability of mHealth development. By evidence-informed or evidence-based HCD practice, they can be interpreted differently in various disciplines. To clarify, this study attempts to investigate pathways to evidence-informed or evidence-based HCD, which is primarily concerned with synthesizing the best-reflective HCD practices. The purpose of this paper is to summarize current HCD practices in mHealth studies and present recommendations supported by a literature review of HCD practice in mHealth research and the reflection from our HCD research experience [18,19]. We hope this paper will inform future research and development of sustainable mHealth apps.

## The Definition of HCD and Its Use to Improve mHealth Adoption

When compared to traditional design methods, incorporating HCD into research methods resulted in more usable, acceptable, and effective health care interventions [20,21]. Numerous definitions of HCD exist and are often specific to the targeted field using HCD [22]. The commonly used International Standardization Organization (ISO) standard ISO 9241-210 [23] definition for HCD suggests that “the human-centered design approach to system’s design and development aims to make interactive systems more usable by focusing on the use of the system and applying human factors/ergonomics and usability knowledge and techniques.” This definition prioritizes building empathy with the target group before attempting to alter or improve their behavior through design improvements [24]. Using HCD and addressing the above requirements afford designers and developers much useful design knowledge to improve the likelihood of mHealth app adoption. HCD differs from user-centered design [25]. The term “user” evolved and was replaced by “human” to humanize the process and emphasize and evoke empathy in the design process [26]. However, in some mHealth studies, the terms “HCD” and “user-centered design” are used interchangeably [27].

## Incorporating Theories and Frameworks in the HCD Practice

The Medical Research Council [28] recommends incorporating stakeholder involvement and the use of theory into the process of complex health intervention development [28]. Various theories and frameworks have been incorporated into HCD practices to develop mHealth apps. These theories are used to address specific aspects of mHealth and have been empirically validated through case studies. The Information Systems Research [29,30], for example, which is developed based on design science and behavior science, has been incorporated as a sociotechnical theory. According to Farao et al [29], current barriers to HCD implementation in health care include combining the needs of end users, HPs, and researchers; differences in approaches taken by designers and researchers; and a lack of evidence from studies that include HCD as a methodology. Importantly, Information Systems Research may improve the use of HCD in the mHealth development process by focusing on rigorous research and data analysis from various sources, accommodating researchers and end-user requirements, and its widespread application in other areas of design [29]. Additionally, Curtis et al [31] synthesized that, while theories and models of behavior change have been used in conjunction with an intervention development framework to develop health interventions, the context in which behavior occurs, reflective process, and explainable change are not specified. To address these challenges, the behavior change wheel framework, a theoretical approach based on the capability opportunity motivation behavior model, was used in conjunction with the HCD concept to develop mHealth apps [31]. The capability opportunity motivation behavior model determines which components must be altered in order for the desired behavior to occur [31]. Another theoretical approach, by Patel and Arya [32], is social marketing, which examines the audience’s perspectives by incorporating product, price, location, and promotion aspects, all of which are important factors to consider when developing mHealth apps. They created the behavior change theories, user-centered design, and social marketing framework by combining behavior change theories, social marketing, and HCD in order to increase the acceptance of design outcomes [32]. In addition to those theories and frameworks that have been incorporated and demonstrated in cases, research also suggests that implementation science could contribute to enhancing the rigor of HCD by incorporating its frameworks, measures, and study designs into the implementation phase of the HCD process [33].

## Common Methods Used in Current HCD Practice

Currently, there are no standardized HCD processes, and HCD practices are reported inconsistently in mHealth studies [29,32,34]. However, similarities exist in these HCD practices. Based on the definition and guiding principles of HCD [23,35-38], we synthesized four major HCD phases commonly referenced in mHealth studies: (1) needs assessment, (2) design and development, (3) laboratory evaluation, and (4) field evaluation. HCD practices have different reporting priorities depending on the research context, with many only reporting on one of the last 2 phases. Various methods were used to assist data collection and analysis in each of these 4 HCD phases. In addition, mixed methods are commonly reported [39]. Often, qualitative research methods are used in the first 2 HCD phases, while mixed methods are usually applied in the third or fourth HCD phases. In Table 1, we summarize the methods used in each HCD phase. Qualitative data collection methods, useful at any stage of the HCD process, included interviews, focus groups, observation, and think-aloud techniques. Quantitative approaches, on the other hand, were more frequently used in the final stages of laboratory and field evaluations. Overall, the most commonly reported methods were self-report questionnaires, such as the poststudy system usability questionnaire [29], the mHealth app usability questionnaire [40], and self-report questionnaires on health outcomes.

**Table 1:** Research methods used at 4 stages.

Stages	Research methods
Need assessment	Observation [29], focus group discussion [31,41], questionnaire [34], persona [34], scenario [34], workshop [42], and qualitative interview
Design and development	Workshop [42] and member check [42]
Laboratory evaluation	Focus group discussion [29], workshop [42], observation [29], think-aloud [29], and self-report questionnaires measuring the usability of mHealth, such as the PSSUQ^a^ [29] and the MAUQ^b^ [40]
Field evaluation	Self-report questionnaires measuring health outcomes, such as the Diabetes Family Responsibility Questionnaire [41]

^a^PSSUQ: poststudy system usability questionnaire.

^b^MAUQ: mHealth app usability questionnaire.

Not surprisingly, mHealth studies used traditional methods that have been widely used in health care research involving patients and HPs. Most HCD studies in mHealth development relied excessively on traditional interviews and focus groups. Both techniques are well suited to pose direct questions but have a limited ability to elicit tacit knowledge [43]. Playing it safe and developing interventions that funders can relate to are more important for applying funding for the intervention development process rather than innovative interventions that require new ways of thinking [44]. However, as mHealth interventions face adoption, efficacy, and sustainability challenges, more dynamic approaches tailored to each mHealth project, as well as more creative methods for improving interaction with end users and other stakeholders, may be needed for mHealth apps to succeed [43].

## Gaps in Current HCD Practice in Achieving Long-Term Efficacy for mHealth Apps

A review of the HCD practice literature specific to mHealth app development indicates five constraints associated with the long-term efficacy of mHealth apps: (1) limited coherence, (2) lack of considerations for long-term viability, (3) undefined measurement, (4) long development time, and (5) publication bias.

### Limited Coherence

Coherence is the degree of consistency when HCD practice is enforced when developing a mHealth app. The incorporation of HCD into the development of mHealth apps necessitates the use of other theories, but they have not yet been developed methodically [29,45,46]. The lack of a theoretical foundation leads to a lack of systematic design procedure, resulting in internally recurrent errors and inefficiency across HCD practices [47]. Many practices have been carried out with the goal of investigating the feasibility of incorporating various theories or theoretical frameworks; however, no follow-up studies have been conducted to provide additional evidence. Limited research exists to address this coherence concern.

### Lack of Considerations for Long-Term Viability

Another major concern is the lack of long-term viability of mHealth apps developed through HCD practice partially due to a different interpretation of outcomes between the design and the public health perspective [27]. For instance, the resulting mHealth app to improve public health may be considered an outcome from a design standpoint, but it is not considered a health outcome from a public health standpoint, where the key outcome of interest is the app’s impact on public health [27]. HCD practice is frequently evaluated through short-term usability testing or short-term feasibility study of design solutions [48]. However, existing mHealth design literature focuses heavily on short-term behavior changes [29] and overlooks long-term usage [48]. Based on the anticipated usage of mHealth apps in lifestyle management, disease management, and even self-diagnosis, desired outcomes are unlikely to be achieved with short-term usage. Emerging research conducted to determine the psychological factors influencing the continued use of mHealth apps emphasizes that the resultful input from users’ perspective is needed in the HCD process to design a long-term mHealth solution [49-51].

### Undefined Measurement

Challenges exist in defining and then selecting measures of success in design outcomes. During the HCD evaluation phase, randomized controlled trials (RCTs) and usability testing may not be sufficient to validate the success of a mHealth app as there are often multiple indirect outcomes that cannot be easily quantified and aggregated (ie, learning and behavioral changes) [52]. Additionally, one-time success is unlikely to provide evidence of the impact on long-term health outcomes. Before adopting a mHealth app, public health and health care practitioners and users usually desire RCT-based evidence that demonstrates the intervention’s effectiveness [44]. However, a conflict often exists between creating an intervention that could be evaluated in an RCT and creating an intervention that can be used in the real-world; efficacy in an RCT often differs from effectiveness in a real-world setting [44].

### Long Development Time

New forms of technology are constantly emerging, and mobile technology is evolving quickly. Meanwhile, valid and reliable mHealth development is always time-consuming, especially the development process with users involved. Therefore, designed mHealth apps should be released in the future, which means they exist outside of the understanding of technology and user paradigms at the time of the HCD process. This can be problematic. Potential solutions for the development team include speculative design, which involves imagining life in the future to create a better-fitting design [53]. However, technology is the result of complex social changes that are difficult to predict. To engage participants in the critical exploration of future technology’s role, forms, and behaviors, creative methods such as user enactment and provotypes were developed and used [54,55]. However, there are still limitations as there is insufficient practice to support speculative methods. More appropriate technology may be developed after the start of the HCD process but before the implementation of mHealth apps, making mHealth less competitive, but the upfront investment is massive.

### Publication Bias

Positive results are more likely to be reported or published, implying publication bias [27]; and this is true in mHealth app development using HCD practice as well. We lack an equal amount of data on the facilitating and inhibiting factors of HCD practice to determine how to make a mHealth solution work. Publication bias limits our ability to learn from and avoids mistakes.

## Recommendations for Developing Sustainable mHealth Apps

### Overview

We reviewed the current HCD practices in mHealth studies and identified interconnected gaps that indicate a lack of comprehensive HCD practices. These gaps in reported HCD practice of mHealth development lead to poor adoption rates and a lack of long-term sustainability. Here, we provide 5 recommendations for future mHealth studies with the goal of developing sustainable mHealth apps using HCD. The recommendations aimed to address the identified gaps and explore the potential of HCD in addressing prevalent challenges with mHealth sustainability. This viewpoint attempts to encourage a discussion toward evidence-informed or evidence-based HCD practice in mHealth development, which can then help address existing gaps in science.

### Consideration of Factors Regarding Culture Norms

Because mHealth design solutions from HCD practice are typically localized, lifestyle-focused, and are part of a system solution, “culture norms” must be considered [56]. In support of this assertion, the digital behavior change intervention framework, for example, stated that cultural norms are an important attribute that influences engagement with digital behavior change interventions by creating different contexts [57]. Current HCD practice in mHealth studies is overly focused on behavior, which might lead to an overlook of how it links to cultural context. Hofstede [58] defined culture as “the collective programming of the mind which distinguishes the members of one human group from another,” and provides 6 dimensions for explaining culture, namely, power distance index, individualism, uncertainty avoidance index, masculinity, long-term orientation, and indulgence versus restraint. In HCD practice, the user needs may be assessed along with these 6 dimensions, particularly when researchers and stakeholders come from different cultural backgrounds. Including cultural aspects helps the mHealth app integration into existing local infrastructure and organizational workflows and results in a more feasible design solution [59]. Cultural aspects have been proven to be an important technology acceptance factor because of their impact on perceived usefulness and perceived ease of use within the technology acceptance model [60]. Research also suggests that behavioral change models including technology acceptance models tend to differ across cultures [61]. Understanding how cultural values influence users’ acceptance of technology is critical for technology designers [47]. Taking cultural norms into account also aids in determining the best manner to communicate with participants during the data collection process of HCD practice [62].

### Iterative Evaluations on HCD Practice

Evaluation of the HCD process and participant experience is just as critical as evaluation of the design solution. However, it is largely unexplored [17]. When building a mHealth app, Bartlett et al [17] analyzed HCD practice and provided useful insights for advanced HCD practice, revealing the importance of HCD practice evaluation. Because HCD practice is iterative, and the data collecting point occurs more than once, it is critical to update the techniques used and conduct process validity throughout the entire HCD practice. We advocate incorporating process validity and satisfaction assessment throughout the HCD practice. Methods such as member checks [63], diary studies [64], focus groups, and questionnaires are suggested.

### Use of Novelty in mHealth App

Novelty is an important aspect to address since lost interest is one of the reasons why the majority of users do not use such apps frequently and may even stop using them for a short time [65]. Gamification, defined as the application of game design features in nongaming circumstances [66], is an effective way to rekindle interest and boost user engagement and has been validated as effective in hypertension-monitoring mHealth app [67]. The theory goes that if interventionists can isolate the active ingredients that make games addictive, they can use them to make digital technologies addictive as well [68]. Because the appropriate gamification differs depending on the target audience and surroundings, the HCD process has the potential to address the key gamification elements for various mHealth contexts. Existing gamification for mHealth includes points, medals, and leaderboards as achievement or progression-oriented gamification elements and social networking as a social-oriented gamification element that can leverage the functions of friending, commenting, and sharing experiences [69]. More research is being performed on creating and deploying gamification design frameworks [70]. It was suggested that HCD can benefit from gamification in sequential work processes [69] and thus enhance mHealth adoption and sustainability.

### Consideration of Privacy and Reliability

Various studies have raised concerns about the quality of mHealth apps, especially the credibility of the medical information in the apps, because some mHealth app development may be driven by developers without professional medical expertise [71]. One of the consequences of unsubstantiated medical information circulation is “infodemic,” defined as “too much information including false or misleading information in digital and physical environments during a disease outbreak which causes confusion and risk-taking behaviors that can harm health” [72]. Same problems arose in mHealth studies using HCD as well. The removal of arbitrary participation of HPs leads to some debate on the reliability of mHealth apps. Some HCD research fails to disclose methods for ensuring up-to-date medical information. HPs and researchers may have a preponderance to have the initiatives on mHealth development to deliver evidence-based care due to their specialized knowledge, networks in the relevant field, and hands-on clinical experience. This is not in opposition to the concept of HCD; rather, the emphasis here is to argue that HPs and researchers are in the best position to organize HCD projects in developing mHealth because the content provided in mHealth apps is critical. HPs and researchers may be able to advance the process of “research into practice,” which is converting promising interventions in clinical research into health care practice [73].

Aside from information reliability, data privacy and security are also of concern. In a cross-sectional analysis of 36 top-ranked apps for depression and smoking cessation available in public app stores, 29 transmitted data to Facebook or Google, but only 12 accurately disclosed this in a privacy policy [74]. Moreover, it is stated that some mHealth policies were developed without references, implying that they are not based on current scientifically obtained facts [75]. This may reflect the power constraint between the municipal, regional, and central levels [75]. Currently, very few studies mentioned that their mHealth apps comply with policies at the national and international levels, resulting in even fewer HCD studies describing how they adhere to current regulations during the design and evaluation phases [76]. Reporting how the HCD project complied with national and international policies for the development of mHealth is essential as it is needed to move forward evidence-based mHealth policies and provide mHealth users a reliable environment.

### Integration of a Sociotechnical Lens

Emery and Trist [77] coined the term sociotechnical systems (STSs) to describe work systems that involve a complex interaction among humans, machines, and the surrounding environment. mHealth apps exist within a complex STS, including a diverse group of people (eg, patients, nurses, and physicians) working with various integrated technologies (eg, mHealth apps and electronic health records) in a dynamic physical and organizational environment [78]. STS emphasizes the involvement of people in the system and focuses on improving the machine-human relationship. Both HCD and STS share the concept of being human-centered, but HCD tends to lead to overreliance on end user input [43], which explains why using the STS lens to demonstrate HCD practice could be beneficial. As van Velsen et al [43] suggested, end user input is commendable, but they are only a subset of the people who should be heard during mHealth design. Ideally, every development team can be seen as an organization [79]. As technology evolves and health care improves, an organization’s ability to adapt to and influence a changing environment determines its sustainability [79]. Because the HCD process is iterative, changes to the work plan occur frequently throughout the development process. Developing a sociotechnical lens to demonstrate HCD practice helps formulate an integrated change strategy.

**Figure 1:**
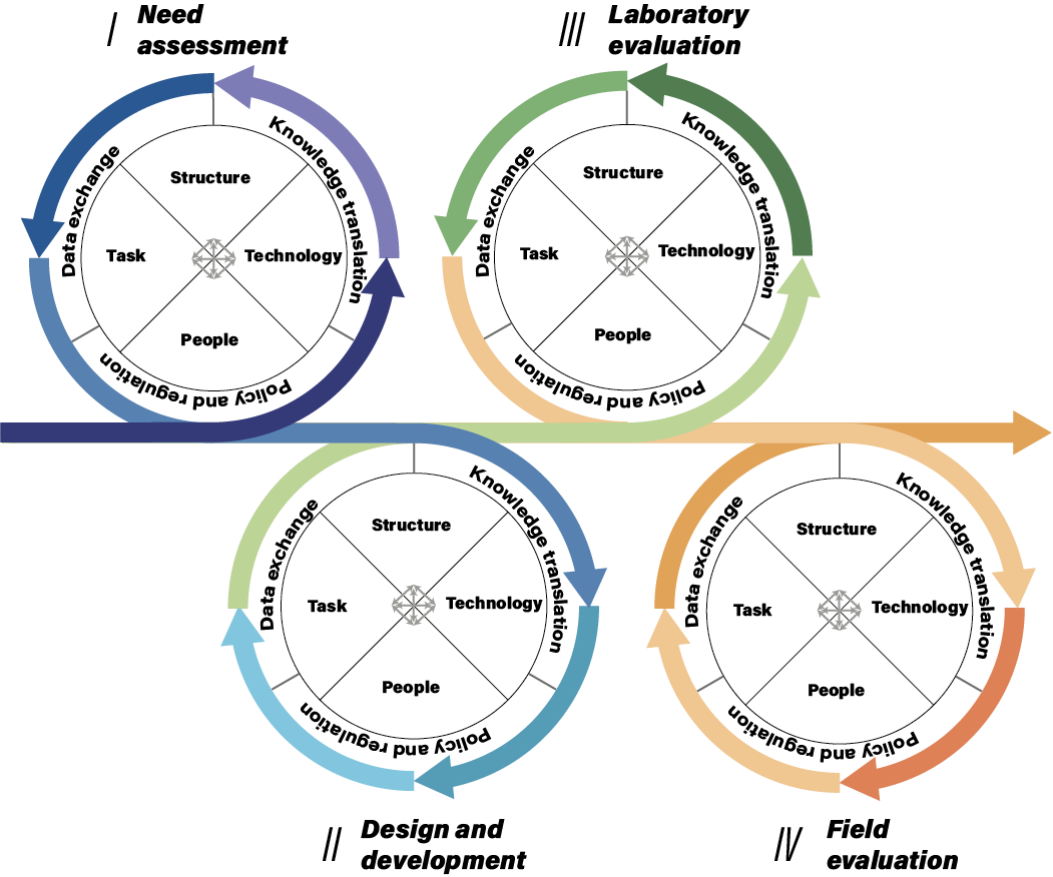
Human-centered design process integrated with a sociotechnical lens.

In Figure 1, we propose a new HCD process with a sociotechnical lens based on an adaptation of Leavitt’s [80] sociotechnical model. By considering the development team as an organization, prior reporting aspects in different phases of HCD practice are located on the 4 dimensions (ie, structure, task, technology, and people). Outside the organizational basis, the external environment includes pluralistic knowledge translation [81], data exchange among institutions, and policy considerations prior to action, all of which need to be considered in the course of the HCD practice. Pluralistic knowledge translation is a dynamic and iterative process that aims to improve the use and usefulness of research in practice [72,82]. The goal of data exchange is to maximize data efficiency while adhering to regulations. Policy and regulations in the field of mHealth development, as well as related fields, should be consulted as early as possible and, if necessary, throughout the process. Additionally, mHealth development may benefit from the concept of sociotechnical imaginaries, which in this case is to envision the compelling future for sustainably developing mHealth initiatives in the STS. Jasanoff and Kim [83] define “sociotechnical imaginaries” as “collectively held, institutionally stabilized, and publicly performed visions of desirable futures, animated by shared understanding of forms of social life and social order attainable through, and supportive of, advances in science and technology.” To date, few rigorous research publications have examined the social, cultural, or political ramifications of eHealth tools. This paper may contribute to the generation of imaginaries of mHealth development and then meanwhile may help to address the development time limitation by conceiving futures.

## Conclusions

mHealth apps have been widely developed and have proven successful, in some instances, in improving patient outcomes and quality of life. However, low adoption and sustainability are known challenges for mHealth apps. In this paper, we reviewed and synthesized the current HCD literature focused on mHealth app development and identified the gaps in current mHealth studies. We offer 5 recommendations for future HCD practice to develop sustainable mHealth apps, which can potentially address the gaps mentioned and contribute to the sustainability of mHealth apps. In particular, a new HCD process with a sociotechnical lens was proposed, which illustrates the integration of HCD phases with an STS in mind. Future research should consider standardizing the HCD practices to help mHealth researchers and developers avoid barriers associated with inadequate HCD practices.

## Abbreviations

HCD: human-centered design
HP: health care provider
ISO: International Standardization Organization
mHealth: mobile health
RCT: randomized controlled trial
STS: sociotechnical system
